# CD34^+^/M-cadherin^+^ Bone Marrow Progenitor Cells Promote Arteriogenesis in Ischemic Hindlimbs of ApoE^−/−^ Mice

**DOI:** 10.1371/journal.pone.0020673

**Published:** 2011-06-03

**Authors:** Toya Terry, Zhiqiang Chen, Richard A. F. Dixon, Peter Vanderslice, Pierre Zoldhelyi, James T. Willerson, Qi Liu

**Affiliations:** 1 The Texas Heart Institute at St. Luke's Episcopal Hospital, Houston, Texas, United States of America; 2 The University of Texas Health Science Center, Houston, Texas, United States of America; Clinica Universidad de Navarra, Spain

## Abstract

**Background:**

Cell-based therapy shows promise in treating peripheral arterial disease (PAD); however, the optimal cell type and long-term efficacy are unknown. In this study, we identified a novel subpopulation of adult progenitor cells positive for CD34 and M-cadherin (CD34^+^/M-cad^+^ BMCs) in mouse and human bone marrow. We also examined the long-lasting therapeutic efficacy of mouse CD34^+^/M-cad^+^ BMCs in restoring blood flow and promoting vascularization in an atherosclerotic mouse model of PAD.

**Methods and Findings:**

Colony-forming cell assays and flow cytometry analysis showed that CD34^+^/M-cad^+^ BMCs have hematopoietic progenitor properties. When delivered intra-arterially into the ischemic hindlimbs of ApoE^−/−^ mice, CD34^+^/M-cad^+^ BMCs alleviated ischemia and significantly improved blood flow compared with CD34^+^/M-cad^−^ BMCs, CD34^−^/M-cad^+^ BMCs, or unselected BMCs. Significantly more arterioles were seen in CD34^+^/M-cad^+^ cell-treated limbs than in any other treatment group 60 days after cell therapy. Furthermore, histologic assessment and morphometric analyses of hindlimbs treated with GFP^+^ CD34^+^/M-cad^+^ cells showed that injected cells incorporated into solid tissue structures at 21 days. Confocal microscopic examination of GFP^+^ CD34^+^/M-cad^+^ cell-treated ischemic legs followed by immunostaining indicated the vascular differentiation of CD34^+^/M-cad^+^ progenitor cells. A cytokine antibody array revealed that CD34^+^/M-cad^+^ cell-conditioned medium contained higher levels of cytokines in a unique pattern, including bFGF, CRG-2, EGF, Flt-3 ligand, IGF-1, SDF-1, and VEGFR-3, than did CD34^+^/M-cad^−^ cell-conditioned medium. The proangiogenic cytokines secreted by CD34^+^/M-cad^+^ cells induced oxygen- and nutrient-depleted endothelial cell sprouting significantly better than CD34^+^/M-cad^−^ cells during hypoxia.

**Conclusion:**

CD34^+^/M-cad^+^ BMCs represent a new progenitor cell type that effectively alleviates hindlimb ischemia in ApoE^−/−^ mice by consistently improving blood flow and promoting arteriogenesis. Additionally, CD34^+^/M-cad^+^ BMCs contribute to microvascular remodeling by differentiating into vascular cells and releasing proangiogenic cytokines and growth factors.

## Introduction

Peripheral arterial disease (PAD) is an atherosclerotic disease that results in insufficient blood flow to the lower extremities [1], [2]. The incidence of PAD, which affects 8 to 12 million Americans, is rapidly increasing, commensurate with the rapid rise in the elderly population [3]. The 2 major clinical stages of PAD are (chronic) intermittent claudication and critical limb ischemia, which can result in loss of the ischemic limb, if not rapidly relieved [4], [5].

Conventional interventional therapy in patients with PAD often results in only partial or short-term correction of lower extremity ischemia. Thus, alternative approaches have been developed, including the local intramuscular injection of autologous adult bone marrow stem/progenitor cells (BMCs) [6], [7], [8]. BMCs engraft into the ischemic target tissues to varying degrees and may alleviate ischemic symptoms [6], [9]. Thus, autologous BMC transplantation has the potential to be a safe alternative treatment strategy for patients with critical limb ischemia for whom current treatment options are not effective [10], [11], [12], [13]. However, initial clinical reports indicated that the significant benefits of BMC therapy are often of limited duration, possibly due to a sharp reduction in the number of locally engrafted progenitor cells early after BMC transplantation [12], [14], [15]. Reduced survival or failed development of transplanted cells may be due in part to cellular atrophy and apoptosis, as well as disorganization and loss of the supporting capillary network in ischemic tissues [14], [16]. The benefits of cell therapy could be strengthened if a donated progenitor cell population retained enriched angiogenic potential and created a pro-angiogenic milieu upon reaching the ischemic area. Thus, these cells would initiate microvessel formation by directly differentiating into vascular cells and by stimulating residual progenitor cell proliferation, mobilization, and engraftment to salvage the compromised tissue.

Accumulating evidence indicates that paracrine action is the predominant mechanism by which progenitor cells contribute to vascular repair and regeneration in ischemic vascular diseases [17], [18]. Experimental reports have shown that conditioned medium (CM) from bone marrow cell cultures contains angiogenic factors, enzymes, and pro-inflammory cytokines that support new blood vessel formation and matrix degradation [19]. However, bone marrow contains a broad array of progenitor and mature cells [20], [21], [22], and the cell type with maximal therapeutic benefits has yet to be identified. Therefore, we seek to identify an optimal bone marrow progenitor cell population that supports extensive vascular regeneration in hindlimb ischemia.

In the present study, we have identified and characterized a population of adult mouse BMCs expressing both CD34 and M-cadherin surface molecules (CD34^+^/M-cad^+^ BMCs), and we have assessed the ability of CD34^+^/M-cad^+^ BMCs to reduce ischemia in a hypercholesterolemic mouse (ApoE^−/−^) hindlimb model during a 60-day postoperative time course. Specifically, we examined the ability to restore blood flow and the arteriogenic potential of CD34^+^/M-cad^+^ BMCs and 3 other related populations: unselected BMCs, a CD34^+^/Mcad^−^ BMC fraction, and a CD34^−^/M-cad^+^ BMC fraction. In addition, we showed the multilineage proliferative potential as well as vascular differentiation of CD34^+^/M-cad^+^ BMCs and assessed the paracrine effects of CD34^+^/M-cad^+^ BMC CM, including its ability to promote endothelial angiogenesis under hypoxia.

## Materials and Methods

### Mouse Ischemic Hindlimb Model and Bone Marrow Cell Transplantation

The University of Texas Health Science Center Animal Welfare Committee guidelines were followed throughout all animal experiments. Mouse BMCs were collected as previously described [23]. ApoE^−/−^ mice (8–15 months old) served as BMC recipients and were anesthetized by isofluorane inhalation (2–5% isofluorane in oxygen) during surgery. By 8 months of age, ApoE^−/−^ mice develop atherosclerotic vascular lesions in peripheral vessels when fed regular chow [24], [25]. Hindlimb ischemia was created by unilateral surgical ligation by using 2 adjacent sutures to interrupt the proximal left femoral artery and vein [23]. To examine the cell population of BMCs that home to ischemic tissues, 2.5×10^7^ unselected BMCs from adult transgenic mice (6–8 weeks old) that expressed an enhanced green fluorescent protein (GFP^+^) variant were injected intraarterially into the ischemic hindlimb of ApoE^−/−^ mice (n = 5) just distal to the ligation site. At 7 days after cell delivery, mice were euthanized, and leg samples were obtained. Additionally, age-matched ApoE^−/−^ mice (n = 5 per group) were divided into 2 groups to evaluate the engraftment of CD34^+^/M-cad^+^ BMCs as compared to CD34^+^/M-cad^−^ BMCs. Ischemia was similarly established, and a single dose of 3×10^5^ GFP^+^ CD34^+^/M-cad^+^ BMCs or CD34^+^/M-cad^−^ BMCs was intraarterially injected into the ischemic hindlimbs; these mice were euthanized at 21 days after cell treatment. Finally, we randomly divided a separate group of ApoE^−/−^ mice into 4 cell treatment groups (n = 9 to 11 mice/group). After undergoing the same surgical procedure described above, the mice received intraarterial injections in the femoral artery of 1 of the following cell types (3×10^5^ cells) isolated from C57BL6/J mice (6–8 weeks old): unselected BMCs, CD34^+^/M-cad^+^ BMCs, CD34^+^/M-cad^−^ BMCs, or CD34^−^/M-cad^+^ BMCs. Mice were euthanized, and leg samples were obtained at 60 days after cell treatment. No immunosuppressant therapy was used during the study.

### Flow Cytometric Analysis and Fluorescence-activated Cell Sorting (FACS)

Freshly isolated BMCs were incubated with fluorescein (FITC)-conjugated anti-mouse CD34 (eBioscience, San Diego, CA), and/or a monoclonal anti-M-cadherin antibody (BD Biosciences, San Diego, CA) for 30 min at 4°C, followed by an additional incubation with the Alexa Fluor-647 goat anti-mouse IgG (Invitrogen, Carlsbad, CA) for 30 min at 4°C. Fluorochrome- and isotype-matched controls (eBioscience and BD Bioscience) were used in parallel experiments to monitor nonspecific staining. The cells were washed, resuspended, and filtered through a 40-µm cell strainer. Propidium iodide was used to exclude nonviable cells. CD34^+^/M-cad^+^ BMCs, CD34^+^/M-cad^−^ BMCs, and CD34^−^/M-cad^+^ BMCs were isolated by using a FACSAir dual-laser fluorescence cell sorter (BD Bioscience). In addition, phycoerythrin-conjugated rat anti-mouse isotype controls and phycoerythrin-conjugated antibodies, including IgG2a, IgG2b, CD31, CD45, and CXCR4 (all from eBioscience), were used to examine the surface antigen profile of CD34^+^/M-cad^+^ BMCs. Data were recorded with a FACS LSRII (BD Biosciences) and analyzed by FACSDiva software.

### Reverse Transcription-Polymerase Chain Reaction (RT-PCR)

RNA isolation was performed according to the protocol provided by the manufacturer (RNeasy Plus Micro Kit, QIAGEN, Germantown, MD). The purity of RNA was estimated by the A_260_/A_280_ ratio (NanoDrop 1000 Spectrophotometer, Thermo Scientific, Waltham, MA). Total RNA from sorted CD34^−^/M-cad^−^, CD34^+^/M-cad^−^, CD34^−^/M-cad^+^, CD34^+^/M-cad^+^ BMC fractions (C57BL6/J mice), C2C12 myoblast cell line (ATCC), human Rh30 cell line (kindly provided by Dr. P. Houghton, Nationwide Children's Hospital), and human CD34^+^ bone marrow mononuclear cells (Stem Cell Technologies, Vancouver, Canada) were reverse transcribed with the oligo dT primer and superscript III first strand synthesis system (Invitrogen). PCR was subsequently carried out with CD34, M-cadherin and GAPDH (internal control) specific primer pairs (Table S1) and platinum Taq DNA polymerase (Invitrogen). The primers were designed by using Primer-Blast provided by NCBI. The sizes of PCR products were determined by comparison to a 100 bp DNA ladder (Invitrogen) under UV light after 1–2% agarose gel electrophoresis.

### Hematopoietic Progenitor Assays

CD34^+^/M-cad^+^ sorted cells were cultured using CollagenCult Medium Kit (StemCell Technologies) per the manufacturer's protocol. The cells were suspended in a medium/collagen solution containing cytokines (10 ng/mL interleukin-3, 10 ng/mL interleukin-6, and 50 ng/mL SCF), and 2.2×10^4^ cells were seeded onto tissue culture plates that were incubated under 5% CO_2_ (37°C at ≥95% humidity). At 21 days, maximum colony size was assessed, and colony-forming cells of the granulocyte/macrophage lineages were identified by morphologic criteria. Collagen gels were dehydrated, and May-Grünwald-Giemsa stains were performed to identify cellular components.

### Laser Doppler Perfusion Imaging

Blood flow in the ischemic (left) and nonischemic contralateral (right) limb was measured by using a laser Doppler perfusion image device (Perimed AB) as previously described [23], and blood flow recovery was determined by comparing flow in the ischemic to nonischemic limb in each mouse [26], [27].

### Arteriolar Density Analysis

Ischemic hindlimbs were decalcified 60 days after cell therapy. Each hindlimb was transversely cut into 5 equal sections (proximal to distal) and embedded in 5 separate paraffin blocks. Arterioles were identified in 6-µm serially cut sections from each tissue block. Sections were stained with a monoclonal antibody directed against α-smooth muscle actin (α-SMA) (1∶50 dilution; Sigma, clone 1A4). Arterioles were identified as vessels that stained positive for α-SMA and had an outer diameter of 10–30 µm. Sections were counterstained with hematoxylin to identify nuclei. Arterioles were counted in a blinded manner in 5 randomly selected high-power fields (520×680 µm) at 20× magnification on transverse sections from each hindlimb. Vessel densities were expressed as the number of arterioles per square millimeter.

### Immunofluorescence Studies

Cell-treated ischemic limbs were decalcified after harvesting at various time points as described above. Frozen leg (treated with 2.5×10^7^ unselected GFP^+^ BMCs for 7 days) cross-sections (6–10 µm) were incubated with anti-M-cadherin antibody (Santa Cruz Biotechnology, Santa Cruz, CA) overnight at 4°C. Sections were then incubated with Alexa Fluor-594 donkey anti-rabbit IgG (Invitrogen, Carlsbad, CA). Nuclei were counterstained with DAPI. Immunostained tissues were visualized with a Leica DM LB fluorescent microscope (Meyer Instruments, Houston, TX). Cryosections (6–10 µm) from hindlimbs (n = 5, 20 sections/leg) treated with 3×10^5^ GFP^+^ CD34^+^/M-cad^+^ BMCs from animals euthanized at 21 days were incubated with primary antibodies against von Willebrand factor (vWF, Abcam), CD146 (Abcam), α-SMA (Abcam), and/or laminin (Sigma). Tissue sections were then incubated with Alexa Fluor-594 donkey anti-rabbit IgG, Alexa Fluor-594 donkey anti-goat IgG, and/or Alexa Fluor-647 donkey anti-rabbit IgG (all from Invitrogen). Nuclei were counterstained with DAPI as above. The fluorescent images were obtained by using a laser scanning confocal microscope (Olympus FV500). Image processing, morphometry, and data analysis were performed by using digital microscopic software SlideBook 5.0 (3i). The correlation of fluorescence intensity in 1 channel with another was measured by using Pearson's correlation.

### Immunohistochemistry

Paraffin-embedded limb sections (6-µm) of ApoE^−/−^ mice (8–12 months old; n = 5) were deparaffinized, rehydrated, and stained with a monoclonal antibody directed against α-SMA (Sigma). Sections were counterstained with hematoxylin to identify nuclei. Vascular images were taken with the use of an inverted light microscope (Olympus IX71) and analyzed with Image-Pro Plus software (Media Cybernetics).

### Cytokine Array

Approximately 2×10^5^ CD34^+^/M-cad^+^ BMCs or CD34^+^/M-cad^−^ BMCs were seeded in separate 10-mm culture dishes in Iscove's Modified Dulbecco's Medium (IMDM, Invitrogen) supplemented with 10% fetal bovine serum (FBS) and 1% penicillin-streptomycin for 24 hrs, and then changed to serum-free IMDM for an additional 24 hrs. Conditioned medium was collected, filtered to eliminate cell debris, and frozen at −80°C. A cytokine array kit was used according to the manufacturer's instructions (RayBiotech, Inc., Norcross, GA). In short, after a brief membrane blocking step, CM was added, and the membrane was incubated overnight at 4°C. After a series of washes, the membrane was incubated with biotinylated anti-cytokine antibodies and horseradish peroxidase conjugated-streptavidin followed by signal detection using ECL-Hyperfilm. Cytokine expression levels were measured using ImageJ software and analyzed with RayBio Analysis Tool S.09 (RayBiotech, Inc).

### Tube and Network Formation Assay

Mouse BMCs were collected as previously described [23] and sorted for CD34^+^/M-cad^+^ and CD34^+^/M-cad^−^ cells. Approximately 7×10^5^ CD34^+^/M-cad^+^ or CD34^+^/M-cad^−^ cells/well were seeded in a 24-well plate with DMEM (10% FBS, 1% PS) and incubated (37°C, 5% CO_2_) overnight. DMEM was then removed, and serum-free DMEM was added to the wells and allowed to incubate for an additional 48 hours. CD34^+^/M-cad^+^ or CD34^+^/M-cad^−^ cell CM was collected and used to perform endothelial tube formation assays.

A collagen-based reduced growth factor membrane matrix (Geltrex™, Invitrogen) was coated (50 µl/cm^2^) onto 24-well plates. A thin-gel method was used to ensure endothelial cell differentiation. The plates were then incubated for 30 minutes to allow the gel to solidify. Afterward, approximately 3.5×10^5^ SVEC4-10 mouse endothelial cells (American Type Culture Collection, Manassas, VA) were resuspended in 200 µl/cm^2^ of CD34^+^/M-cad^+^ CM or CD34^+^/M-cad^−^ CM with the addition of 1% FBS final concentration. SVEC4-10 cells were seeded into 24-well plates and placed in an incubator (37°C, 20% O_2_ 5% CO_2_) to allow tube formation to develop. Tube formation was documented every hour until a pronounced difference between CD34^+^/M-cad^+^ or CD34^+^/M-cad^−^ cell CM group was observed. Additionally, we performed the tube formation assay with SVEC4-10 cells under hypoxic conditions (37°C, 1% O_2_, 5% CO_2_). The cells were placed in serum-free media for 24 hours in a hypoxia chamber (Galaxy 14S, New Brunswick, Edison, NJ). After starvation, cells were prepared as above, and tube formation was carried out in the hypoxia chamber. Tube formation was documented at 4, 6, and 8 hours incubation.

Digital images were taken using an inverted light microscope (Olympus IX71, Leeds Instruments, Irving, TX) at a 40× magnification for all assay conditions. Assays were performed in triplicate; images were taken in 3 different microscopic fields/well, and the total cumulative tube length (µm) was measured. Analysis was performed by using NIH Image J software.

### Statistical Analysis

Data were expressed as mean ± standard error of mean (SEM). One-way analysis of variance (ANOVA) with the Mann-Whitney *post hoc* test was used to determine statistical significance within and between groups (GraphPad Prism 5). *P*<0.05 was considered statistically significant.

## Results

### Mouse Bone Marrow Contains a Population of Double-Positive CD34^+^/M-cad^+^ Cells

To identify a subpopulation of BMCs that targets ischemic tissues, unselected BMCs from GFP^+^ mice were intraarterially injected into the ischemic hindlimbs of ApoE^−/−^ mice. After 7 days, transverse sections of the ischemic hindlimbs were analyzed by using immunofluorescence. A panel of antibodies was used to detect engrafted cells derived from donated GFP^+^ BMCs. During these initial investigations, we found that GFP^+^ cells that co-expressed M-cadherin localized to the damaged tissue area, as shown in Figure S1. Previously, we reported the detection of donated GFP^+^ BMCs co-expressing CD34 in treated ischemic limbs [23].

**Figure 1:**
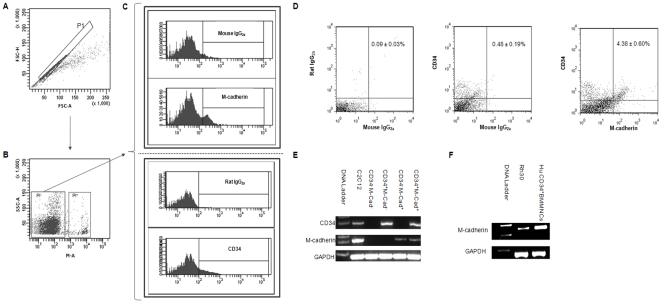
Identification of CD34^+^/M-cadherin^+^ bone marrow progenitor cells. (**A**) Scatter plot of BMCs collected from C57BL6/J mice. The P1 gate was used to discriminate doublets. (**B**) BMC viability. Propidium iodium (PI) was used to exclude dead cells (PI^+^ cells) from further analysis. (**C**) Identification of M-cad^+^ and CD34^+^ subsets. Indirect staining with M-cadherin antibody revealed a small fraction of M-cad^+^ BMCs compared with isotype-and fluorochrome-matched controls. CD34^+^ BMCs were detected by an FITC-conjugated anti-CD34 monoclonal antibody. (**D**) Identification of a CD34^+^/M-cadherin^+^ subpopulation from freshly isolated BMCs. (**E**) RT-PCR analysis of CD34 and M-cadherin mRNA in sorted mouse BMC subpopulations. (**F**) The detection of M-cadherin mRNA in human CD34^+^ BMMNCs by RT-PCR.

After conducting immunostaining experiments to identify GFP^+^ CD34^+^ cells and GFP^+^ M-cad^+^ in the ischemic limbs of ApoE^−/−^ mice following GFP^+^ BMC treatment, we further investigated CD34 and M-cadherin expression in bone marrow cells by flow cytometry. To ensure specific and accurate detection, isotype- and fluorochrome-matched controls to CD34 and M-cad antibodies were used in parallel. Flow cytometric analysis of freshly isolated viable BMCs (Fig. 1A and 1B) that were stained with M-cad antibody (Fig. 1C) showed a small subpopulation of M-cad^+^ cells (5.30±0.63%, n = 4; Fig. 1C) as compared to those that stained positive with mouse isotype control IgG2a (1.42±0.29%). Analysis also revealed an expected CD34^+^ BMC population (8.86±0.58%). In addition, a distinct double-positive population of CD34^+^/M-cad^+^ BMCs (4.28±0.60%, Fig. 1D) was identified by flow cytometry after costaining BMCs with CD34 and M-cad antibodies. For additional validation, the expression of CD34 and M-cadherin on sorted CD34^+^/M-cad^+^ BMCs was confirmed by RT-PCR. The mouse myoblast cell line, C2C12, known for expressing both CD34 and M-cadherin [28], was used as a positive control during RT-PCR (Fig. 1E). Furthermore, M-cadherin expression in CD34^+^ human bone marrow mononuclear cells (BMMNCs) was studied by RT-PCR. Using specific human M-cad primer pairs (Table S1), we detected a definite PCR product indicating the existence of M-cad transcript in CD34^+^ BMMNCs (Fig. 1F). An M-cadherin–expressing human Rh30 cell line was used as positive control [29]. Finally, immunophenotyping of surface-antigen profile indicated that mouse CD34^+^/M-cad^+^ BMCs possess hematopoietic characteristics with high (≥85%) expression levels of CD45, CD31, and CXCR4 (Fig. S2).

**Figure 2:**
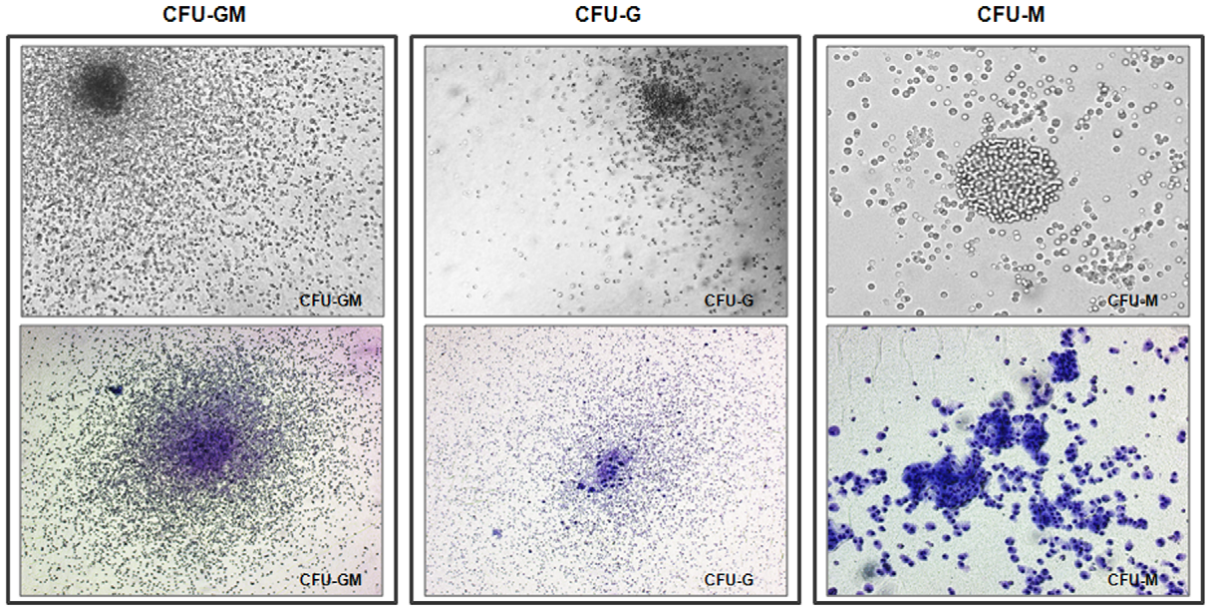
CD34^+^/M-cad^+^ BMCs give rise to hematopoietic progenitors. Representative photomicrographs of CD34^+^/M-cad^+^ BMCs reveal both granulocytic and monocytic lineages (CFU-GM, CFU-G, and CFU-M). Top row, photomicrographs taken under inverted light microscope; bottom row, May-Grünwald-Giemsa stain. Magnification: 40×, CFU-GM and CFU-G; 100×, CFU-M.

The multipotent clonogenic potential of CD34^+^/M-cadherin^+^ BMCs population was examined in colony-forming unit (CFU) assays. Figure 2 shows the proliferative potential and enriched hematopoietic colony-forming activity (ie, CFU-G, CFU-M and CFU-GM) of CD34^+^/M-cadherin^+^ BMCs grown in collagen-based semi-solid medium supplemented with SCF, IL-3, and IL-6. This finding suggests that this cell type is a hematopoietic progenitor population with multilineage development capability.

### Treatment With CD34^+^/M-cad^+^ Cells Significantly Improves Recovery of Persistent Blood Flow in the Ischemic Hindlimbs of ApoE^−/−^ Mice

**Figure 3:**
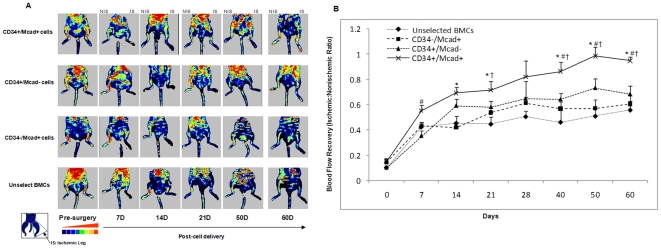
Recovery of blood flow in the ischemic limbs of ApoE^−/−^mice treated with CD34^+^/M-cad^+^ BMCs. (**A**) Representative laser Doppler perfusion images taken at indicated intervals for the 4 treatment groups (n = 9–11/group). Blood perfusion in the ischemic hindlimbs was markedly increased in the CD34^+^/M-cad^+^ BMC group compared with other cell treatment groups. IS = ischemic legs, NIS = nonischemic legs. (**B**) Quantitative analysis of hindlimb blood perfusion. The increase in ischemic∶nonischemic ratio was significantly higher in the CD34^+^/M-cad^+^ BMC group compared with other treatment groups at multiple time points. Data are expressed as mean ± SEM, n = 9–11/group. *P<0.01, CD34^+^/Mcad^+^ vs unselected BMC group; ^#^ *P*<0.05, CD34^+^/Mcad^+^ vs CD34^+^/Mcad^−^; ^†^ *P*<0.05, CD34^+^/Mcad^+^ vs CD34^−^/Mcad.^+^ Maximal blood flow perfusion was set at 1.0 compared to the contralateral leg after femoral artery ligation.

We compared the efficacy of sorted CD34^+^/M-cad^+^ BMCs to that of CD34^+^/M-cad^−^ cells, CD34^−^/M-cad^+^ cells, and unselected BMCs in restoring blood flow to ischemic hindlimbs. Blood flow was measured before treatment, 30 minutes after treatment, and every 7–10 days thereafter up to 60 days. Representative laser Doppler images (Fig. 3A) illustrate perfusion of the ischemic (left) legs versus the nonischemic contralateral limbs. As early as 7 days after surgery, blood flow recovery was better in mice treated with CD34^+^/M-cad^+^ BMCs than in any other treatment group. Moreover, flow recovery was significantly better in CD34^+^/M-cad^+^ BMC–treated mice than in mice treated with CD34^+^/M-cad^−^ BMCs at 7 days (55.44±4.31% vs 35.38±4.32%; *P*<0.05; n = 9–11; Fig. 3B). At 14 and 21 days, the CD34^+^/M-cad^+^ BMC treatment group showed significantly superior blood flow recovery over unselected BMCs (*P*<0.01). At 21 days, the CD34^+^/M-cad^+^ BMC treatment group showed improvement over the CD34^−^/M-cad^+^ treatment group (*P*<0.05). Importantly, mice treated with CD34^+^/M-cad^+^ BMCs showed significantly improved blood flow over CD34^+^/M-cad^−^ (*P*<0.05), CD34^−^/M-cad^+^ (*P*<0.05), and unselected (*P*<0.01) BMCs at 40, 50, and 60 days. Furthermore, 10–30% of the mice from the other treatment groups developed necrotic hindlimbs or died within 60 days as compared with none in the CD34^+^/M-cad^+^ cell treatment group (Table S2). These findings indicate that CD34^+^/M-cad^+^ treatment reduced ischemia-related muscle necrosis.

### CD34^+^/M-cad^+^ Cell Therapy Results in Significantly Greater Arteriogenesis in the Ischemic Legs of ApoE^−/−^ Mice

**Figure 4:**
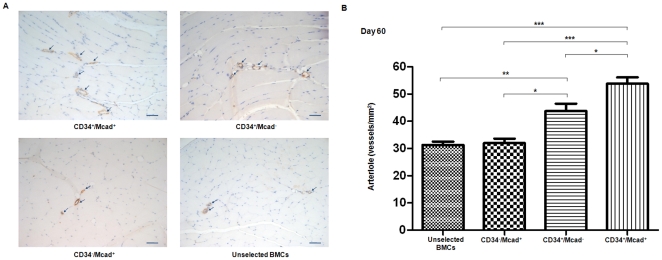
Significant arteriogenesis in ischemic limbs of ApoE^−/−^ mice after treatment with CD34^+^/M-cad^+^ BMCs. ApoE^−/−^ mice were euthanized at 60 days after cell treatment. Transverse sections (6 µm) were immunostained against α-smooth muscle actin and counterstained with hemotoxylin. (**A**) Representative photomicrographs of arterioles (arrows) in ischemic legs treated with the indicated cell type. Scale bar = 50 µm. (**B**) Summary of quantitative analysis of arteriole density in 4 treatment groups at 60 days after cell treatment. Values are mean ± SEM, n = 4/group;*P<0.01, **P<0.001, ***P<0.0001.

Because neovascularization is believed to be essential for maintaining perfusion recovery, we studied arteriogenesis after cell therapy. To identify arteries and arterioloes, we immunostained tissue sections of ischemic legs with α-SMA 60 days after cell treatment. Arterioles (luminal size 10–30 µm) were quantified in a blinded manner. We analyzed 408 sections from the 4 treatment groups (average, 105–130 sections/group; n = 4) and found significantly increased arteriogenesis in the CD34^+^/M-cad^+^–treated group compared to mice treated with CD34^+^/M-cad^−^ cells, CD34^−^/M-cad^+^ cells, or unselected BMCs cells (*P*<0.01, *P*<0.001, *P*<0.0001, respectively; Fig. 4). The identification and our analysis of arterioles from 5 different anatomic levels throughout the entire leg provide evidence of widespread neovascularization promoted by treatment with CD34^+^/M-cad^+^ BMCs.

### CD34^+^/M-cad^+^ BMCs Engraft into Solid Tissues and Differentiate into Vascular Structures Under Ischemic Conditions

**Figure 5:**
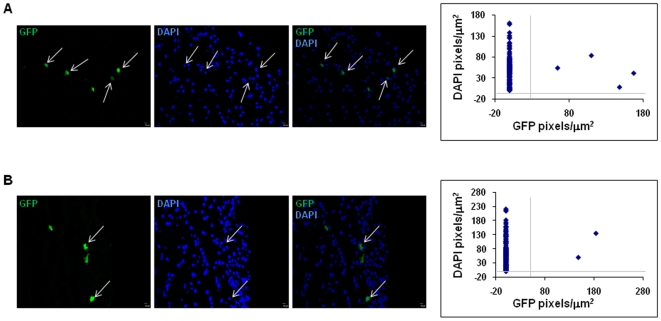
Recruitment of GFP^+^ CD34^+^/M-cad^+^ and GFP^+^ CD34^+^/M-cad^−^ BMCs at 21 days after cell therapy. Representative confocal micrographs and quantitative analysis show GFP^+^ cells colocalized with DAPI in the ischemic legs of ApoE^−/−^ mice treated with (**A**) GFP^+^ CD34^+^/M-cad^+^ BMCs and (**B**) GFP^+^ CD34^+^/M-cad^−^ BMCs. N = 5 ApoE^−/−^ mice/cell treatment group. Scale bar = 10 µm.

**Figure 6:**
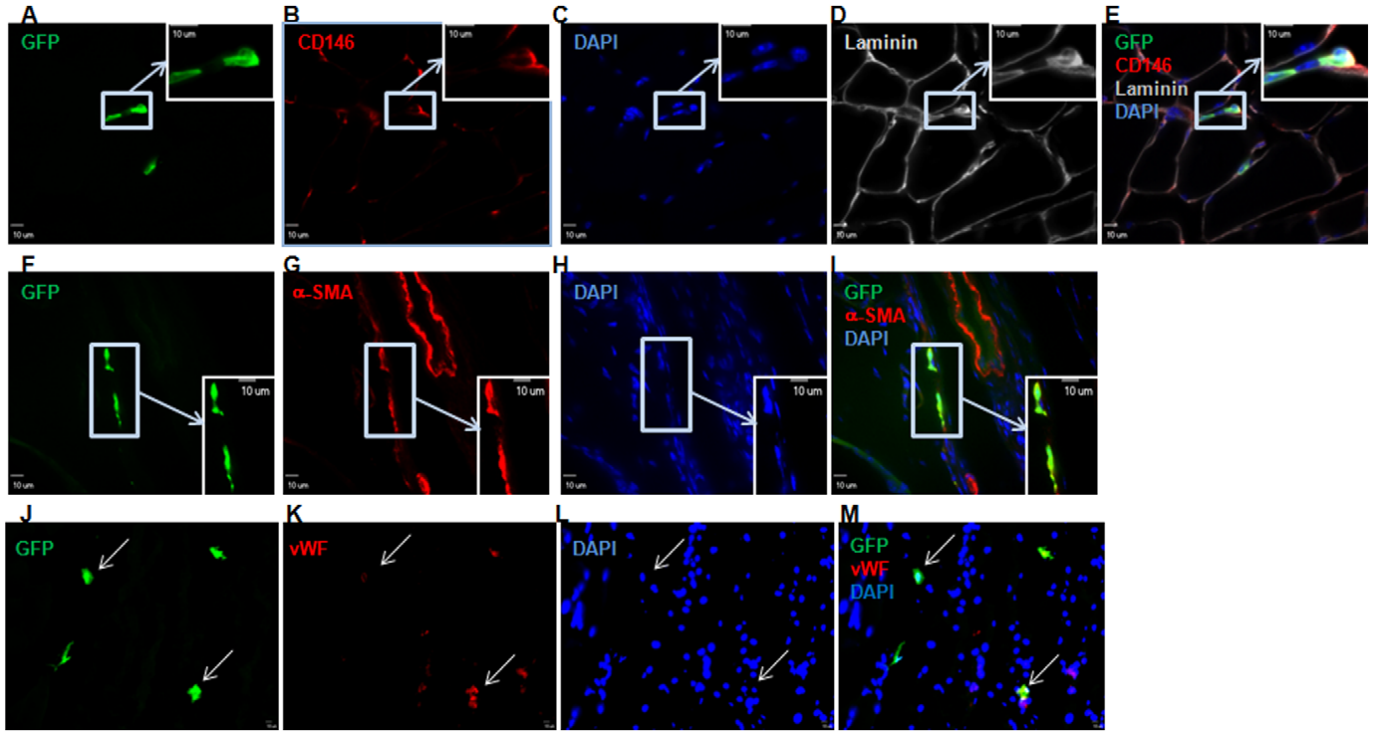
Vascular development of GFP^+^ CD34^+^/M-cad^+^ BMCs in the ischemic legs of ApoE^−/−^ mice. ApoE^−/−^ mice were euthanized 21 days after GFP^+^ CD34^+^/M-cad^+^ cell therapy, and the cell-treated legs were processed. Immunofluroescence staining for CD146, α-SMA, and von Willebrand Factor (vWF) was used to identify endothelial and smooth muscle cells that originated from GFP^+^ CD34^+^/M-cad^+^ BMCs in cross-sections of muscle tissue. (**A, F, and J**) Confocal images of muscle cross-sections showing the presence of GFP^+^ cellular structures. (**B, G, and K**) CD146, α-SMA, and vWF immunostaining. (**C, H, and L**) DAPI nuclei counterstaining. (**D**) Myofibers outlined by laminin staining. (**E**) Merged image of A, B, C, and D. GFP^+^ cells coexpressing CD146 are displayed in the insert. (**I**) Merged image of F, G, and H. GFP^+^ cells colocalized with α-SMA as shown in the enlarged image, indicating vasculature formation from transplanted cells. (**M**) Merged image of J, K, and L. Arrows indicate colocalization of GFP and vWF-positive cells.

To examine whether CD34^+^/M-cad^+^ progenitor cells incorporate into hindlimbs and differentiate into vascular cells, we delivered a single dose of 3×10^5^ GFP^+^ CD34^+^/M-cad^+^ BMCs into the ischemic legs of ApoE^−/−^ mice and euthanized the mice 21 days after cell delivery. Long-term engraftment of injected cells was assessed histologically on transverse sections of cell-treated legs. To measure the GFP^+^ area (pixels/µm^2^) in cross-sections of leg samples, we masked the individual nuclei in entire laser scanning confocal images of the specimen. Morphometric analysis in combination with Pearson's correlation was used to quantify the colocalization of GFP expression with nuclei (DAPI) signals. We determined that authentic GFP^+^ cells incorporated into recipient mouse legs (Fig. 5A). The same experimental analysis procedures were applied to specimens treated with GFP^+^ CD34^+^/M-cad^−^ cells (Fig. 5B). However, we observed considerable differences in the distribution of GFP^+^ cells in anatomic areas, partially due to the delivery route (intrafemoral delivery of donor cells) and to a low level of engraftment (<1%). Thus, we were unable to calculate the accurate engraftment rate of donated GFP^+^ cells. Despite the limited incorporation of donated progenitor cells, confocal microscopic examination of transverse leg sections followed by CD146 (endothelial cell marker) or α-SMA staining revealed vascular differentiation of transplanted GFP^+^ CD34^+^/M-cad^+^ cells in ischemic legs at 21 days (n = 5). Donated GFP^+^ CD34^+^/M-cad^+^ progenitors gave rise to endothelial and smooth muscle structures in the muscle tissue of ApoE^−/−^ mice (Fig. 6). Using confocal microscopic analysis of cross-sections of limb muscular tissues followed by immunofluorescence staining with the endothelial marker vWF, we found colocalization of GFP with vWF signals (Fig. 6), confirming the differentiation of GFP^+^ CD34^+^/M-cad^+^ cells into endothelial cells. Thus, our data clearly demonstrated that CD34^+^/M-cad^+^ progenitors can integrate into host structures and serve as common vascular progenitors for postnatal arteriogenesis. Although GFP^+^ CD34^+^/M-cad^+^ cells integrated into vascular structures, we were unable to find functional arterioles or arteries that originated entirely from GFP^+^ CD34^+^/M-cad^+^ cells. Limited incorporation of GFP^+^ CD34^+^/M-cad^+^ cells into functional arterioles makes direct vascular differentiation of this cell type into newly formed arterioles an unlikely major mechanism that mediates arteriogenesis.

### CD34^+^/M-cad^+^ BMCs Secrete a Unique Array of Cytokines and Growth Factors

**Figure 7:**
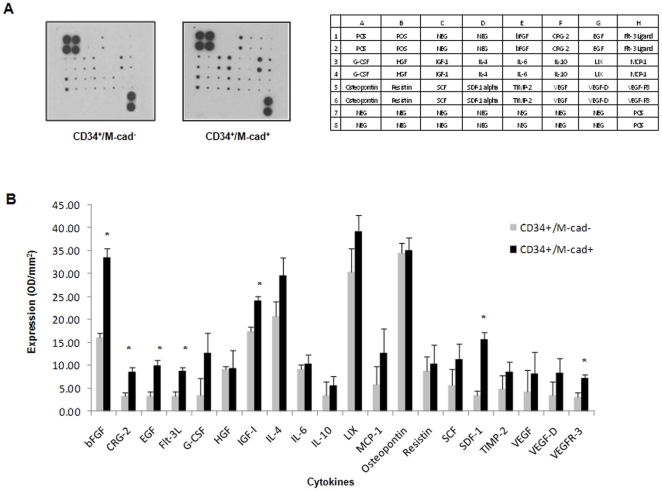
Increased cytokine secretion from CD34^+^/M-cad^+^ BMCs compared to CD34^+^/M-cad^−^ BMCs. (**A**) Representative membranes and corresponding analysis key for CM from each indicated cell population. (**B**) CM from CD34^+^/M-cad^+^ BMCs showed significant secretion levels of key stimulatory cytokines compared with CM from CD34^+^/M-cad^−^ BMCs. The cytokine arrays (n = 3) were quantified by using the S.09 analysis tool (RayBiotech, Inc). Data are expressed as mean ± SEM. *P<0.05 vs CM of CD34^+^/M-cad^−^ BMCs. bFGF, basic fibroblast growth factor; CRG-2, cytokine responsive gene-2; EGF, epidermal growth factor; Flt-3L, FMS-like tyrosine kinase-3 ligand; G-CSF, granulocyte colony stimulating factor; HGF, hepatocyte growth factor; IGF-1, insulin-like growth factor-1; IL, interleukin; LIX, lipopolysaccharide inducible CXC chemokine; MCP-1, monocyte chemoattractant protein-1, SCF, stem cell factor; SDF-1, stromal cell-derived factor-1; TIMP-2, tissue inhibitor of metalloproteinases-2; VEGF, vascular endothelial growth factor.

Since the benefits of adult stem/progenitor cell therapy, including angiogenesis, arteriogenesis and vasculogenesis, are mainly derived from the paracrine effects of transplanted cells [17], [19], we assessed and compared the in vitro release of cytokines and growth factors by CD34^+^/M-cad^+^ and CD34^+^/M-cad^−^ BMCs. We quantified the secretion of multiple proangiogenic and proliferative factors by using a cytokine antibody array (Fig. 7). Of the 20 cytokines measured, basic fibroblast growth factor (bFGF), epidermal growth factor (EGF), and insulin-like growth factor-1 (IGF-1) were significantly increased in the CM of CD34^+^/M-cad^+^ cells as compared to CM from CD34^+^/M-cad^−^ BMCs. Additionally, CD34^+^/M-cad^+^ cells released significantly higher levels of Flt-3 ligand (Flt-3L), a growth factor involved in survival, proliferation, and differentiation of early progenitor cells and endothelial vessels [30]. Furthermore, the CM of CD34^+^/M-cad^+^ cells had significantly higher levels of SDF-1, which along with its specific receptor CXC chemokine receptor 4 (CXCR4), plays a critical role in promoting progenitor cell trafficking, engraftment, and angiogenesis [31], [32]. Levels of CXCL-10 (CRG-2), which recruits human mesenchymal stem cells (MSCs) [33], were significantly higher in CD34^+^/M-cad^+^ CM than in CM of CD34^+^/M-cad^−^ BMCs. Together, our results provide clear evidence that CD34^+^/M-cad^+^ cells secrete a variety of prosurvival and proangiogenic cytokines that can induce a functional vascular network.

### Conditioned Medium from CD34^+^/M-cad^+^ Cells Enhances Angiogenesis In Vitro Under Hypoxic Conditions

**Figure 8:**
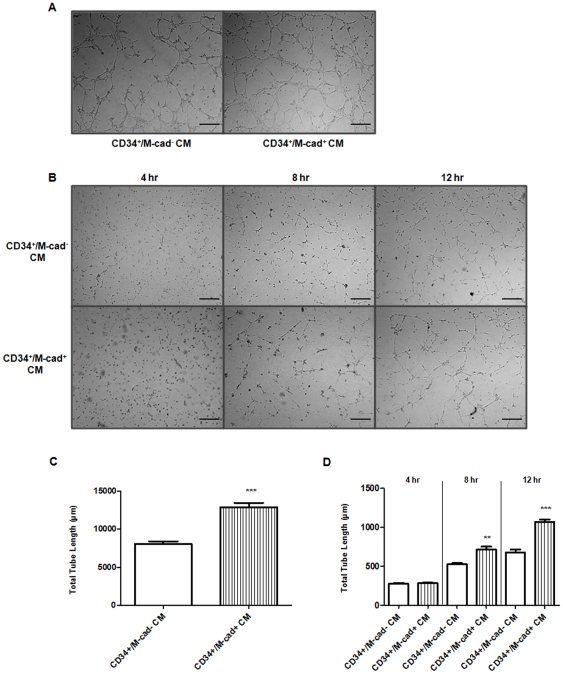
CD34^+^/M-cad^+^ CM enhanced endothelial cell capillary tube network formation. (**A**) Serum-starved SVEC4-10 cells were mixed with collagen matrix and incubated with CD34^+^/M-cad^+^ or CD34^+^/M-cad^−^ CM under normal (20%O_2_, 5%CO_2_, 37°C) conditions for 4 hours. (**B**) Hypoxic (1%O_2_, 5%CO_2_, 37°C), serum-starved SVEC4-10 cells were mixed with collagen matrix and incubated with CD34^+^/M-cad^+^ -or CD34^+^/M-cad^−^ CM for up to 12 hrs. Timed-incubation revealed that capillary-like tube formation was greater in the CD34^+^/M-cad^+^ CM–treated group than in the CD34^+^/M-cad^−^ CM–treated group. Scale bar = 50 µm. (**C**) Quantification of tube length in SVEC4-10 cells after incubation with CD34^+^/M-cad^−^ or CD34^+^/M-cad^+^ CM under normoxic conditions (n = 3,***P<0.001 vs CD34^+^/M-cad^−^). (**D**) Quantification of tube length in SVEC4-10 cells at 4, 8, and 12 hours after incubation with CD34^+^/M-cad^−^ or CD34^+^/M-cad^+^ CM under hypoxic conditions (n = 3, **P<0.01, ***P<0.001 vs CD34^+^/M-cad^−^). Total tube length (µm) was reported as mean ± SEM.

We used a common reliable tube formation assay to examine the ability of CD34^+^/M-cad^+^ cells to drive angiogenesis of endothelial cells in vitro. Nutrient-starved mouse endothelial cells (SVEC4-10) incubated with CD34^+^/M-cad^+^ or CD34^+^/M-cad^−^ CM under normoxic conditions (20%O_2_, 5%CO_2_, 37°C) effectively migrated towards each other, directionally aligned themselves, and formed a complex tubular network as early as 4 hours after seeding on the matrix (Fig. 8A). However, the total length of the tubes was significantly increased in endothelial cells incubated with CD34^+^/M-cad^+^ CM when compared to tube length in endothelial cells incubated with CD34^+^/M-cad^−^ CM (Fig. 8C).

To further test the angiogenic effects of CD34^+^/M-cad^+^ CM versus CD34^+^/M-cad^−^ CM during ischemia, we preconditioned nutrient-starved mouse endothelial cells under hypoxic conditions (1%O_2_, 5%CO_2_, 37°C) for 24 hours prior to incubating them with individual CM. In the presence of CD34^+^/M-cad^+^ or CD34^+^/M-cad^−^ CM, tubular structures were created. However, fewer capillary-like networks were formed and longer incubation period were required as compared with endothelial cells treated with the same CM during normoxia (Fig. 8B). Under hypoxic conditions, the total tube length of endothelial cells treated with CD34^+^/M-cad^+^ CM was significantly higher at 8 and 12 hours than the tube length seen in endothelial cells treated with CD34^+^/M-cad^−^ CM (Fig. 8D). These findings are consistent with our in vivo results showing significant restoration of blood flow perfusion and greater arteriogenesis in the ischemic legs of ApoE^−/−^ mice.

## Discussion

We have identified a novel population of CD34^+^/M-cad^+^ hematopoietic progenitor cells in adult mouse bone marrow that improved blood flow recovery better than CD34^+^/M-cad^−^ and CD34^−^/M-cad^+^ cells in an atherosclerotic mouse model of PAD. Significant blood flow recovery was maintained for up to 60 days after CD34^+^/M-cad^+^ cell treatment. Furthermore, CD34^+^/M-cad^+^ BMCs promoted uniform neoformation of arteriolar vessels during a 60-day period. The pro-angiogenic property of CD34^+^/M-cad^+^ cells was facilitated by their direct regeneration of endothelial and smooth muscle cells in ischemic muscles. Multiple proangiogenic cytokines/growth factors released from the transplanted CD34^+^/M-cad^+^ cells may synergistically augment vasculogenic effects as well. Furthermore, we have shown the existence of CD34^+^/M-cad^+^ population in human BMMNCs, making this population a potential candidate for clinical use.

An animal model that resembles human PAD is crucial for examining and validating the potential benefits of any novel cell type in a preclinical study. PAD resulting from atherosclerosis produces chronic ischemia. Mice that are deficient in apolipoprotein E (ApoE^−/−^) develop spontaneous atherosclerosis that narrows the vessel lumen, which leads to progressive restriction of blood flow at multiple arterial branches [24], [34], [35]. Reddick and colleagues analyzed in detail the age-dependent progressive increase in atherosclerosis in ApoE^−/−^ mice fed normal mouse chow [24]. Complex, extensive atherosclerotic lesions (eg, foam cells, cholesterol clefts, fibrous-cap lesions, and calcification) and severe vessel occlusion were observed by 8 months of age. These lesions were found not only in the aorta and the heart, but also in the peripheral vessels, including the iliac arteries. Our findings support those of Reddick and colleagues [24] in that we observed spontaneous atheromas in the hindlimb vessels of 8–12–month-old ApoE^−/−^ mice (n = 5) fed normal chow (Fig. S3). The atherosclerotic lesions had several histologic characteristics that significantly narrowed or damaged the vessels (Fig. S3). Therefore, we used 8–15–month-old ApoE^−/−^ mice as cell therapy recipients because they develop chronic atherosclerosis similar to the atherosclerotic lesions seen in human [36], [37].

CD34^+^ BMCs have been shown in animal and human studies to possess primitive hematopoietic and endothelial progenitor cell potential [38], [39], [40], [41]. M-cadherin belongs to the cadherin multigene family of transmembrane glycoproteins [42]. As a calcium-dependent cell adhesion molecule, M-cadherin is involved in cell-cell communication and skeletal muscle development [42], [43]. Expression of M-cadherin and CD34 has been detected in subsets of satellite cells that are able to repair injured muscle [28], [44]. Corti and colleagues isolated M-cad^+^ BMCs from freshly harvested mouse bone marrow cells that expressed myogenic markers and showed myogenic potential after delivery into injured muscle tissue [45]. Although the study by Corti and coauthors focused on the potential repair of muscle injury by bone marrow-derived myogenic cells, we demonstrated the beneficial effect of CD34^+^/Mcad^+^ BMCs in the treatment of hindlimb ischemia via the enhancement of neovascularization. The myogenic potential of CD34^+^/M-cad^+^ BMCs should be further studied as our ultimate goal is to apply cell therapy to promote angiogenesis and myogenesis concomitantly in the ischemic limbs.

In this study, we demonstrated the superior ability of CD34^+^/M-cad^+^ BMCs to enhance postischemic arteriogenesis compared to CD34^+^/M-cad^−^ BMCs, CD34^−^/M-cad^+^ BMCs, and unselected BMCs. The comprehensive physiologic role of CD34^+^/M-cad^+^ BMCs may contribute to their direct vascular differentiation and their trophic effect on intrinsic stem cell activation. Evidence indicates that BMCs produce an array of proinflammatory cytokines and proangiogenic factors capable of enhancing angiogenesis [18]. In the present study, we found significantly higher levels of growth factors, including bFGF, EGF, and IGF-1, in the CM of CD34^+^/M-cad^+^ BMCs than in the CM of CD34^+^/M-cad^−^. Moreover, higher levels of bFGF were released by CD34^+^/M-cad^+^ BMCs than by CD34^+^/M-cad^−^ BMCs (Fig. 7B), suggesting that bFGF may contribute to the differential therapeutic effects observed with CD34^+^/M-cad^+^ cells. EGF, another well-described growth factor, is noted for its vasculogenic influence on stem cells. Kilroy and colleagues found that stem cells supplemented with EGF doubled their secretion of HGF in a synergistic fashion to promote additional growth and cellular preconditioning, helping to minimize damage after ischemic injury [46]. IGF-1 has been shown to enhance muscle regeneration by activating myogenic stem/progenitor cells [47], [48]. Sustained dual delivery of IGF-1 and the proangiogenic factor VEGF into ischemic hindlimbs resulted in a synergistic effect on angiogenesis and myogenesis [49]. Our results also suggest the beneficial interplay among cytokines secreted by CD34^+^/M-cad^+^ BMCs. The preferential release of IGF-1 and proangiogenic factors (eg, bFGF) may account for the persistent vascular effects in the ischemic legs of ApoE^−/−^ mice.

In addition to those described above, we found that CD34^+^/M-cad^+^ progenitor cells released several unique angiogenic and vasculogenic cytokines. The CM of CD34^+^/M-cad^+^ cells showed significant levels of Flt-3L, a tyrosine kinase associated with stimulation of early hematopoietic cell differentiation [30]. Furthermore, we found that the stimulatory cytokines SDF-1 and CXCL-10 (CRG-2) were also significantly increased in the CM of CD34^+^/M-cad^+^ BMCs compared to those of CD34^+^/M-cad^−^ CM. CXCL-10 (CRG-2) stimulates the migration and recruitment of MSCs [33]. A significant release of CXCL-10 by CD34^+^/M-cad^+^ BMCs may help recruit endogenous MSCs from the bone marrow to assist in the recovery of blood flow and synergize with cytokines to promote arteriogenesis. Interestingly, the soluble form of VEGFR3 (sVEGFR3) was markedly upregulated in our cytokine analysis of the CM of CD34^+^/M-cad^+^ BMCs. VEGF receptor3 (VEGFR3) is expressed in lymphatic vessels under physiologic conditions [50]. sVEGFR3 inhibits VEGFR3 signaling by binding to its ligands, VEGF-C and VEGF-D, which results in a reduction of lymphangiogenesis [51]. In a model of transgenic mice expressing sVEGFRs, Makinen and colleagues showed that the effect of sVEGFR3 was specific to the lymphatic vessels and did not affect the blood vessel network [51]. In animal tumor models, overexpression of VEGFR3 has been found in tumor metastasis. By inhibiting VEGF-C and VEGF-D signaling, sVEGFR3 sustained tumor growth and suppressed metastasis formation [52]. Our array analysis showed a higher secretion of sVEGFR3 (although at a low total level) by CD34^+^/M-cad^+^ cells than by CD34^+^/M-cad^−^ cells; considering our array profile data, this finding may indicate that the delicate balance of a cocktail of factors released by progenitor cells is vital for promoting therapeutic arteriogenesis and preventing malignant growth. Further examination of paracrine-mediated arteriogenesis may lead to enhanced strategies for cell therapy.

The aim of the present study was not to assess the benefit of a single factor secreted by CD34^+^/M-cad^+^ cells, but rather to examine whether the pleiotropic activity of this population can enhance vascular repair and new vessel growth in ischemic tissue. The novelty and advantage of using CD34^+^/M-cad^+^ cells is due to their vascular differentiation capability, their secretion of a distinct combination of angiogenic and myogenic factors, their ability to rescue compromised cells in a hypoxic environment, and their substantial in vivo neovascularization capacity. These beneficial effects position CD34^+^/M-cad^+^ cells as an optimal cell type for treating ischemic vascular diseases such as PAD, where local cells are oxygen- and nutrient-deprived from the shortage of blood. Because our cytokine array was restricted to the simultaneous measurement of only 20 cytokines in CD34^+^/M-cad^+^ cell supernatant, a large-scale study may uncover additional proangiogenic cytokines.

In conclusion, we have described a novel adult subpopulation of BMCs–CD34^+^/M-cad^+^cells–that promotes widespread arteriolar growth and persistent blood flow recovery in the ischemic hindlimbs of immunocompetent ApoE^−/−^ mice. This population displays hematopoietic proliferative potential, gives rise to vascular cells, secretes a unique pattern of cytokines and growth factors, and can significantly stimulate angiogenesis in nutrient- and oxygen-depleted endothelial cells under hypoxic conditions. Our results provide evidence that CD34^+^/M-cad^+^ cells can protect against ischemic injury and may be a suitable candidate for use in cell therapy against PAD.

## Supporting Information

Figure S1 **Identification of M-cad^+^ BMCs in the ischemic legs of ApoE^−/−^ mice injected with GFP^+^ BMCs.** (**A**) Immunostaining for M-cad in engrafted cells, 7 days after intraarterial injection of unselected GFP^+^ BMCs. (**B**) DAPI counterstaining. (**C**) Merged image of A and B. Scale bar = 20 µm. N = 5 ApoE^−/−^ mice.
(TIF)

Figure S2 **Flow cytometric characterization of the surface antigens of CD34^+^/M-cad^+^ BMCs.** Representative analysis figures show high expression levels of CD31, CXCR4, and CD45 in CD34^+^/M-cad^+^ BMCs.
(TIF)

Figure S3 **Atherosclerosis in hindlimb vessels of ApoE^−/−^ mice.** Representative α-smooth muscle actin immunostaining images reveal spontaneous atherosclerotic lesions (indicated by arrows) in the hindlimb arteries of ApoE^−/−^ mice (8–12 months old). (**A**) Plaque within the peripheral artery. (**B**) Medial hypertrophy. Enlarged images are shown in boxes.
(TIF)

Table S1 **Primers used for RT-PCR.**
(TIF)

Table S2 **Clinical symptoms in ApoE−/− mice after cell therapy.**
(TIF)
