# Supplementary figures and images for: CD34+/M-cadherin+ Bone Marrow Progenitor Cells Promote Arteriogenesis in Ischemic Hindlimbs of ApoE−/− Mice
Source: PLoS One. 2011 Jun 3;6(6):e20673. doi: 10.1371/journal.pone.0020673 (PMC3108984; doi:10.1371/journal.pone.0020673)

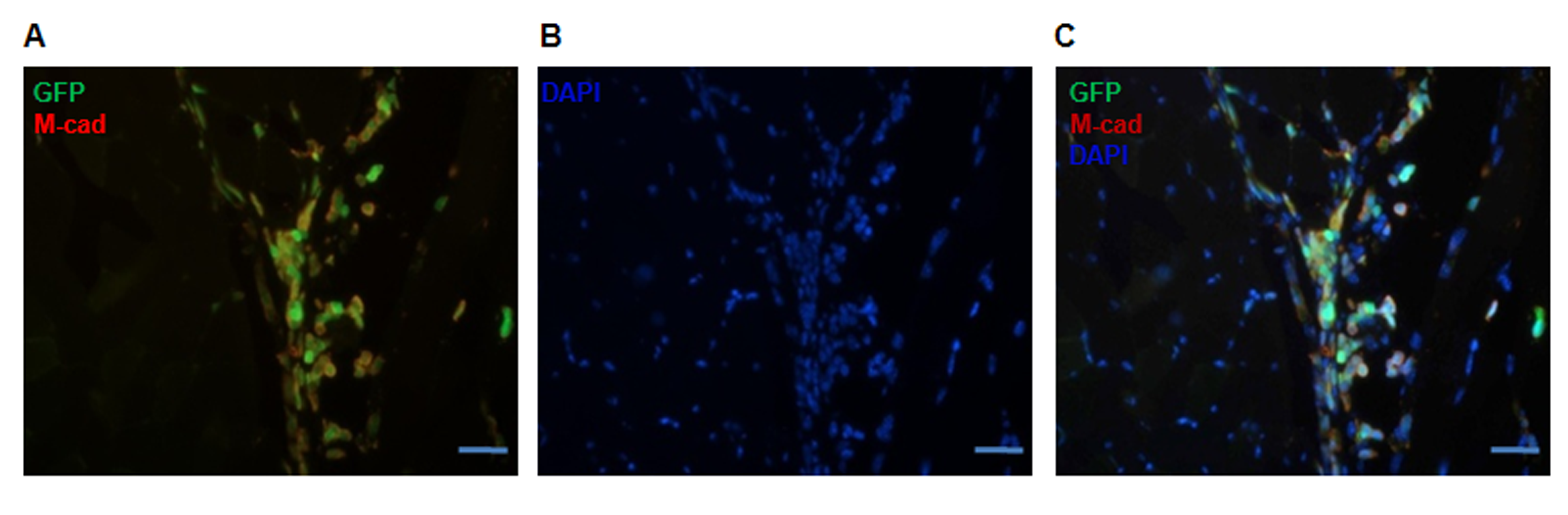

Supplement: Figure S1 — Identification of M-cad+ BMCs in the ischemic legs of ApoE−/− mice injected with GFP+ BMCs. (A) Immunostaining for M-cad in engrafted cells, 7 days after intraarterial injection of unselected GFP+ BMCs. (B) DAPI counterstaining. (C) Merged image of A and B. Scale bar = 20 µm. N = 5 ApoE−/− mice. (TIF) [file pone.0020673.s001.tif]

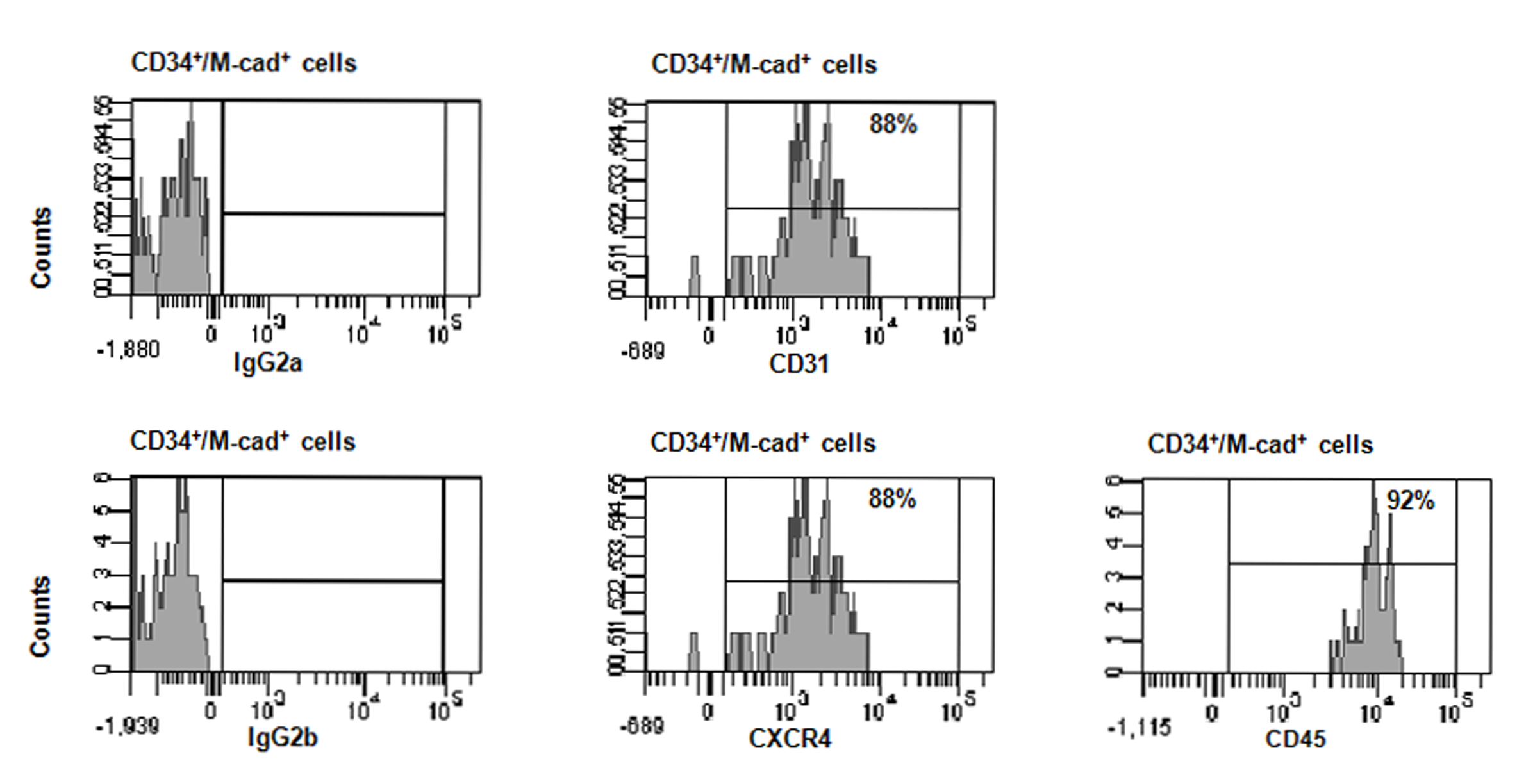

Supplement: Figure S2 — Flow cytometric characterization of the surface antigens of CD34+/M-cad+ BMCs. Representative analysis figures show high expression levels of CD31, CXCR4, and CD45 in CD34+/M-cad+ BMCs. (TIF) [file pone.0020673.s002.tif]

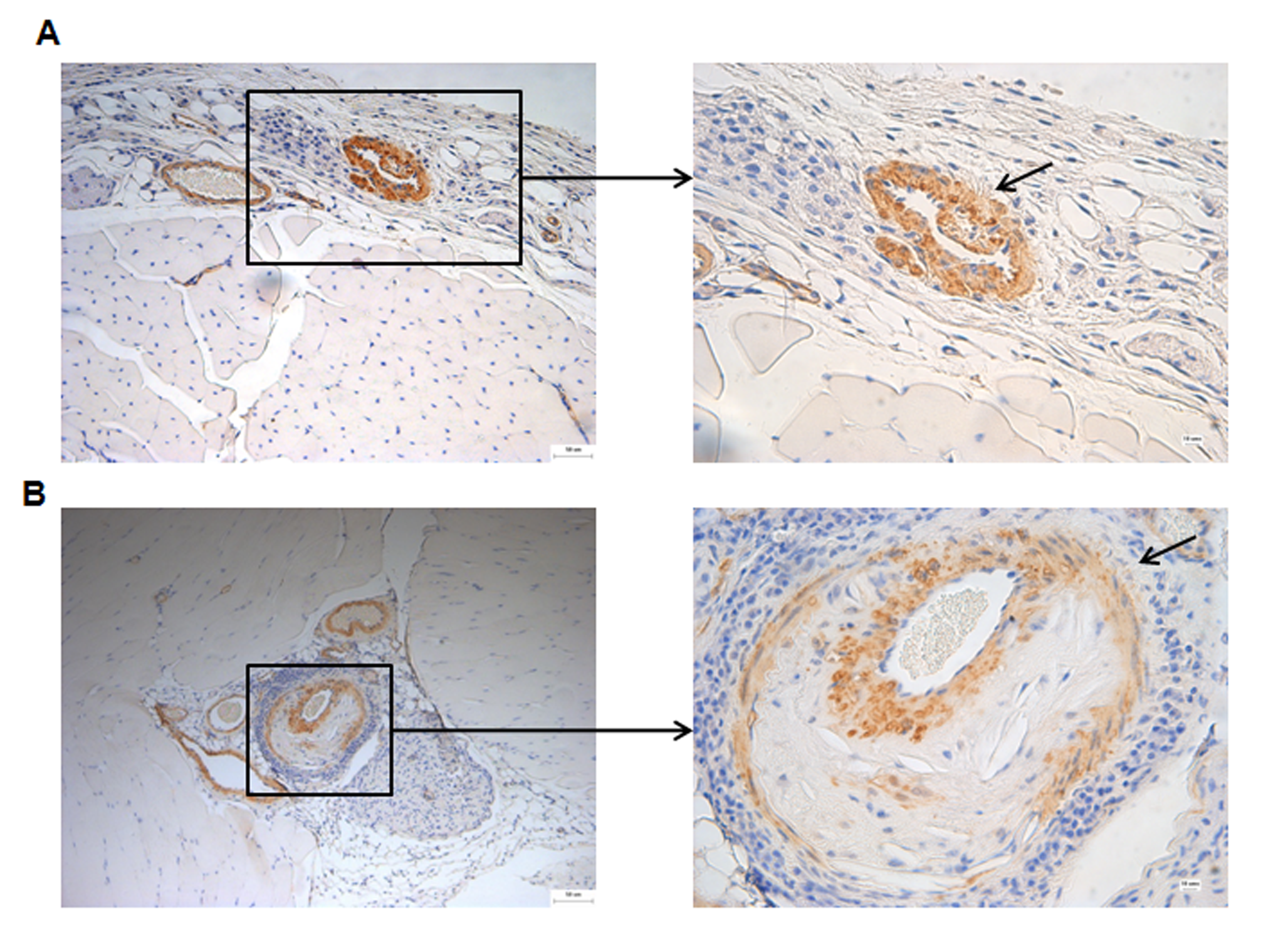

Supplement: Figure S3 — Atherosclerosis in hindlimb vessels of ApoE−/− mice. Representative α-smooth muscle actin immunostaining images reveal spontaneous atherosclerotic lesions (indicated by arrows) in the hindlimb arteries of ApoE−/− mice (8–12 months old). (A) Plaque within the peripheral artery. (B) Medial hypertrophy. Enlarged images are shown in boxes. (TIF) [file pone.0020673.s003.tif]

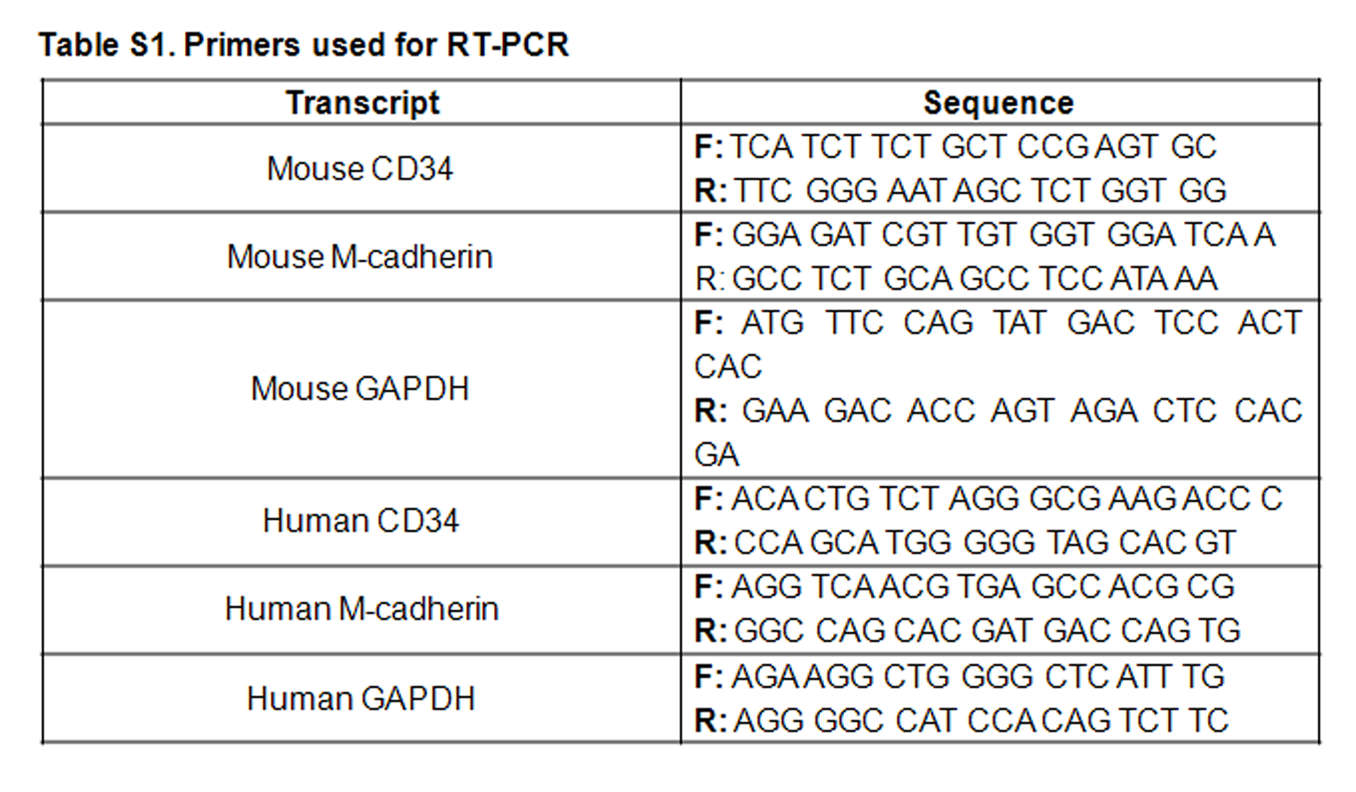

Supplement: Table S1 — Primers used for RT-PCR. (TIF) [file pone.0020673.s004.tif]

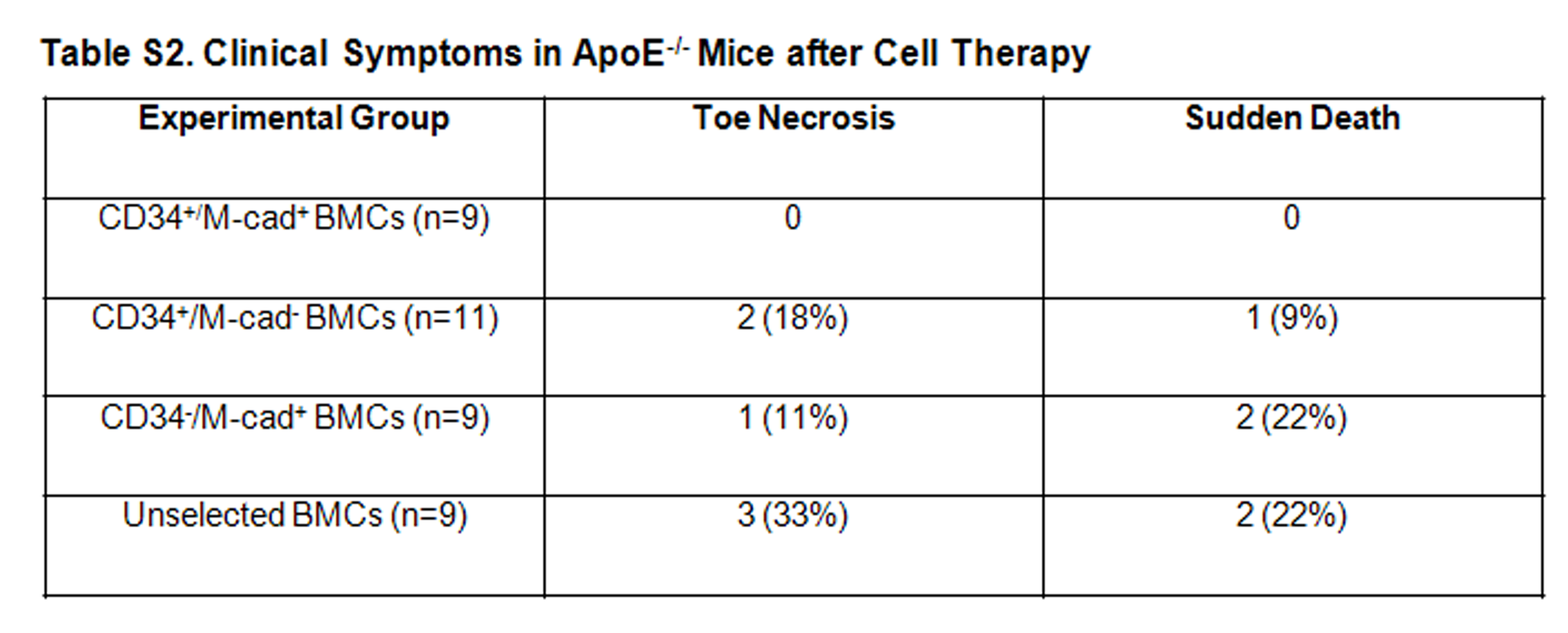

Supplement: Table S2 — Clinical symptoms in ApoE−/− mice after cell therapy. (TIF) [file pone.0020673.s005.tif]
